# Ultrasound - based deep learning radiomics nomogram for noninvasive prediction of p53 mutation status in hepatocellular carcinoma: a variational autoencoder based development and validation study

**DOI:** 10.3389/fonc.2026.1756941

**Published:** 2026-04-29

**Authors:** Shuangxi Chen, Xushuang Qin, Shanni Dong, Jiamei Qiu, Yuting Liu, Yang Liu, Li Zhu, Fengzhi Li

**Affiliations:** 1 Cancer Center, Department of Ultrasound Medicine, Zhejiang Provincial People’s Hospital, Affiliated People’s Hospital, Hangzhou Medical College, Hangzhou, Zhejiang, China; 2 The Second School of Clinical Medicine, Hangzhou Normal University, Hangzhou, Zhejiang, China

**Keywords:** deep learning, hepatocellular carcinoma, p53, radiomics, VAE

## Abstract

**Objectives:**

Accurate preoperative assessment of p53 mutation status in hepatocellular carcinoma (HCC) is critical for prognostic stratification and personalized treatment planning. Conventional radiomics approaches often suffer from feature redundancy and limited generalization. This study aimed to develop and validate a noninvasive ultrasound-based radiomics nomogram integrating variational autoencoder (VAE)-derived deep features to predict p53 expression status, addressing these limitations.

**Methods:**

A retrospective cohort of 172 patients with pathologically confirmed HCC (training cohort: n=120, validation cohort: n=52) who underwent preoperative two-dimensional ultrasound images and had available p53 immunohistochemistry (IHC) results was analyzed. ultrasound images were segmented, and radiomic features were extracted from them. A VAE network was employed to reduce feature dimensionality and extract high-level malignant risk scores. These scores were integrated with clinical variables (Alpha-Fetoprotein [AFP] levels, Microvascular Invasion [MVI] status, and Edmondson-Steiner (E-S) grade) to construct a predictive nomogram. Model performance was evaluated using receiver operating characteristic (ROC) analysis (area under the curve [AUC]), calibration curves, and decision curve analysis (DCA).

**Results:**

The VAE-integrated nomogram achieved robust predictive performance, with an AUC of 0.925 (95% CI: 0.881–0.969) in the training cohort and 0.820 (95% CI: 0.699–0.942) in the validation cohort. Calibration curves demonstrated close alignment between predicted and observed probabilities, and decision curve analysis confirmed clinical utility across a broad threshold probability range. Key clinical benefits included noninvasive assessment of p53 mutation and enhanced interpretability through combined deep learning and clinical parameters.

**Conclusion:**

This VAE-based radiomics framework effectively combines deep feature representation with clinical variables, providing a reliable tool for noninvasive preoperative evaluation of HCC p53 mutation. The model shows promise for optimizing surgical decision-making and personalized prognostic strategies in HCC management.

## Introduction

Hepatocellular carcinoma (HCC) ranks as the third-leading cause of cancer-related deaths globally, with its five-year survival rate below 20% due to frequent recurrence and tumor heterogeneity (1, 2). p53, a pivotal tumor suppressor governing cell cycle progression and apoptosis, has emerged as a critical prognostic biomarker, where overexpression correlates strongly with aggressive phenotypes, early postoperative recurrence, and reduced overall survival in HCC (3, 4). While preoperative assessment of p53 status could inform surgical planning and adjuvant therapy selection, current dependence on invasive biopsy limits its clinical utility and precludes longitudinal monitoring.

Advances in radiomics have enabled non-invasive tumor phenotyping through high-dimensional feature extraction from medical imaging (5, 6). Nevertheless, three critical limitations persist: First, conventional feature selection methods (e.g., Least Absolute Shrinkage and Selection Operator (LASSO) and Minimum Redundancy Maximum Relevance (mRMR)) often fail to capture nonlinear feature interactions, resulting in redundant feature sets. Second, model generalizability remains suboptimal, with performance degradation observed in external validation cohorts. Third, most studies neglect the synergistic integration of deep radiomic representations with clinicopathological determinants.

To address these gaps, we propose a variational autoencoder (VAE)-driven radiomic framework. Unlike deterministic dimensionality reduction techniques, VAEs probabilistically encode high-dimensional radiomic features into latent representations while reconstructing synthetic data to enhance model robustness against overfitting (7, 8). This architecture inherently mitigates feature redundancy by learning compressed embeddings of ultrasound image texture patterns and facilitates cross-institutional generalizability through synthetic data augmentation (9). Building upon these advantages, our study developed a VAE-based nomogram that synergistically integrates latent radiomic features with clinical indicators AFP and microvascular invasion (MVI) to optimize the predictive performance for p53 overexpression in HCC. This multimodal approach provides a non-invasive imaging biomarker for screening HCC patients who may benefit from targeted therapy or immunotherapy regimens.

## Materials and methods

### Study population

This retrospective cohort study was approved by the Institutional Ethics Committee of Zhejiang Provincial People’s Hospital (Approval No.: QT2025045). The requirement for written informed consent was waived due to the retrospective observational design.

To protect patient privacy, all the data were desensitized before use, and relevant prescribed guidelines were implemented in this study.

**Figure 1:**
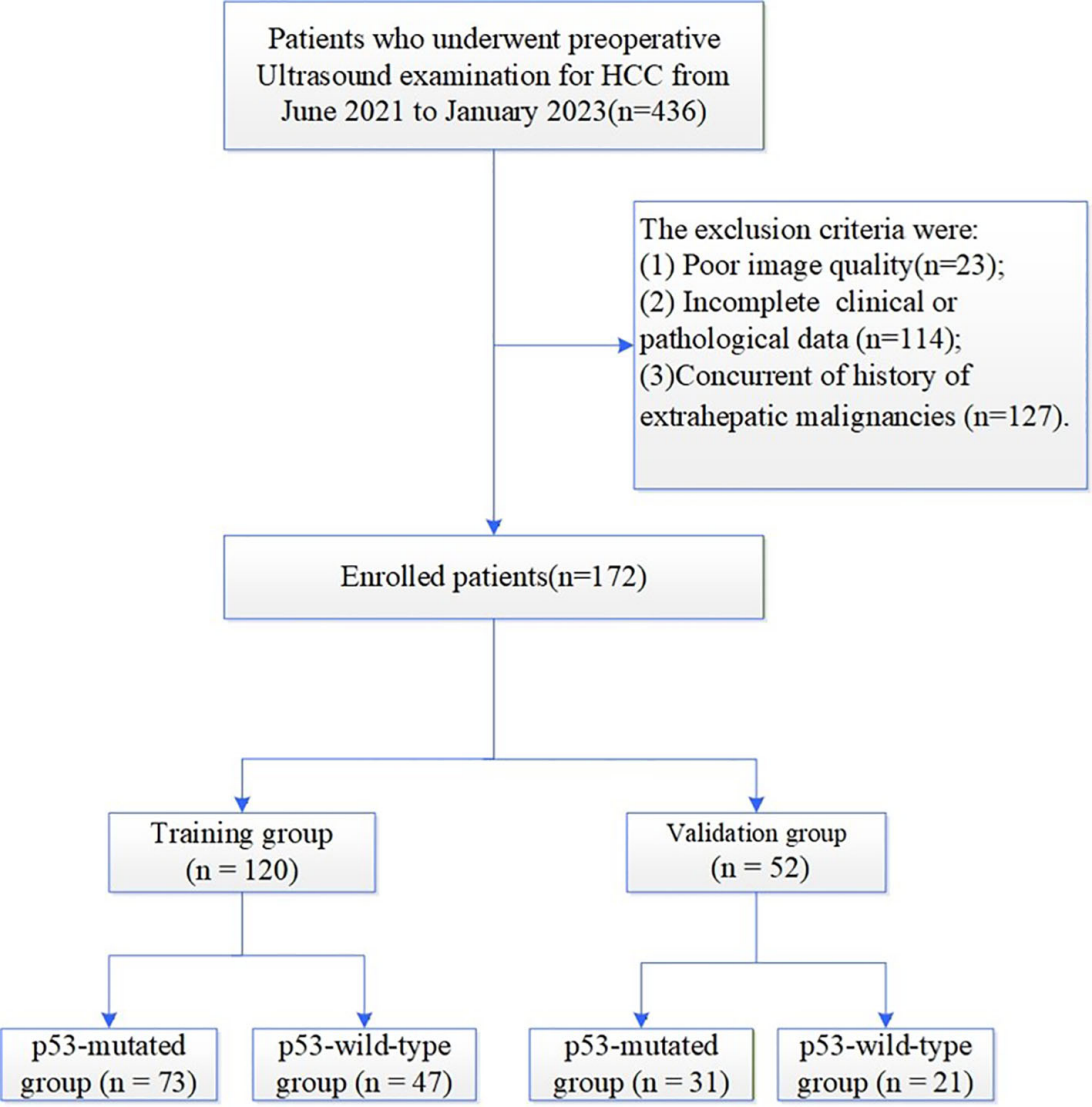
Flowchart of participant enrollment.

Between June 2021 and January 2023, 436 consecutive patients who underwent surgical resection with histopathologically confirmed HCC were initially screened. Inclusion criteria comprised: (1) age ≥18 years; (2) Child-Pugh class A/B liver function; (3) availability of histopathological data including p53 mutation status, MVI, and Edmondson-Steiner grading; (4) preoperative liver ultrasound examination performed ≤1 week before surgery; (5) no prior anticancer therapy (e.g., chemotherapy, radiotherapy, or targeted therapy); (6) for multifocal HCC, selection of the dominant lesion corroborated by both histology and immunohistochemistry. Exclusion criteria included: (1)poor image quality (n =23); (2) incomplete clinical or pathological data (n=114); (3) concurrent or history of extrahepatic malignancies (n=127). Following exclusions (**Figure 1**, study flowchart), 172 eligible patients were enrolled. The cohort was randomly allocated into a training cohort (n=120, 70%) for model development and a validation cohort (n=52, 30%) for performance evaluation, preserving a 7:3 ratio to ensure statistical robustness.

### Liver ultrasound examination

All ultrasound examinations were performed using color Doppler systems (GE LOGIQ E20, Mindray Resona R9) equipped with abdominal transducers (1–5 MHz). Transabdominal scanning was employed to acquire comprehensive imaging of hepatic tumors. During transabdominal examination, patients were positioned in the supine position. The ultrasound features assessed included tumor size, presence of cirrhosis, tumor capsule, tumor margin, and intratumoral necrosis.

### Baseline clinical and pathological variables

Demographic, tumor-related, and clinicopathological data were retrospectively extracted from institutional electronic medical records and systematically categorized as follows:

Demographics: Age (continuous variable), sex (male/female). Virological status: serum hepatitis B surface antigen (HBsAg+/-). Serological biomarkers: AFP: Dichotomized at 20 μg/L (clinically relevant cutoff for HCC surveillance per EASL guidelines); Liver function parameters: Total bilirubin (TBIL, μmol/L), albumin (ALB, g/L), prothrombin time (PT, seconds). Clinical staging: Child-Pugh classification (A/B); Barcelona Clinic Liver Cancer (BCLC) stage (0/A/B/C). Histopathological features: MVI: present/absent, Edmondson-Steiner (E-S) grade (I–IV).

### Ultrasound image analysis

The ultrasound features assessed included tumor size, presence of cirrhosis, tumor capsule, tumor margin, and intratumoral necrosis. All ultrasound images were initially reviewed independently by an abdominal ultrasonography physician with 12 years of experience and subsequently validated and categorized by another abdominal ultrasonography physician with 16 years of experience. In cases of disagreement, a third abdominal ultrasonography physician with 25 years of experience was consulted until a consensus was reached to determine the final diagnosis. Throughout this process, all three abdominal ultrasonography physicians were blinded to the clinical and pathological information of the patients to ensure the objectivity and independence of the evaluation.

### Pathological examination

p53 immunohistochemical (IHC) staining was independently evaluated by two board−certified pathologists with ≥8 years of experience in liver cancer pathology, focusing on the proportion and intensity of nuclear staining in tumor cells. A mutant−type pattern was defined as strong, diffuse nuclear positivity involving ≥70% of tumor nuclei, reflecting abnormal protein accumulation due to conformational stabilization from missense mutations; a wild−type pattern was defined as weak or focal staining in ≤10% of nuclei or complete absence of staining, consistent with the short half−life of native p53 protein. Cases exhibiting heterogeneous expression (10%–70% positive nuclei) were subjected to confirmatory DNA sequencing to verify mutation status. This standardized assessment enabled reliable classification of p53 status, facilitating integration with imaging phenotypes and downstream clinical correlation analyzes.

### Tumor segmentation and radiomics feature extraction

Preoperative ultrasound images were retrieved in Digital Imaging and Communications in Medicine (DICOM) format and processed using the open-source segmentation software ITK-SNAP (v3.8.0; http://www.itksnap.org). Regions of interest (ROI) encompassing the entire tumor volume were manually delineated along tumor boundaries on axial slices by two board-certified abdominal radiologists (with 9 and 16 years of clinical experience, respectively). In patients with multifocal lesions, the dominant mass exhibiting the maximum cross-sectional diameter was selected for subsequent analysis. Radiomic feature extraction was performed via PyRadiomics (v3.0.1), yielding 1,316 quantitative imaging biomarkers across three categories: (1) First-order intensity statistics (e.g., energy, entropy, kurtosis); (2) Second-order textural parameters derived from gray-level co-occurrence (GLCM) and run-length (GLRLM) matrices; and (3) Higher-order features incorporating wavelet-transformed morphological characteristics. All computational procedures adhered to the Image Biomarker Standardization Initiative (IBSI) guidelines to ensure cross-platform reproducibility.

### Feature selection and model construction

In this retrospective study involving 172 patients, radiomics features were comprehensively preprocessed before analysis. Continuous variables were scaled to the interval 0-1 (-1~1) using min-max normalization, and outliers were handled by median imputation. For clinical variables, dimensionality reduction was performed using logistic regression to enhance feature interpretability and reduce the risk of overfitting.

We employed a Variational Autoencoder (VAE) approach to select key radiomics features. A five-layer VAE architecture was constructed to select radiomics features through latent space representation learning. The network configuration was as follows: Input layer: 1316 normalized radiomics features; Encoder path: three fully connected layers with neuron counts of 128 → 32 → 16; Activation functions: ReLU for the first four layers and PReLU for the output layer; Loss function: MSELoss, comprising a classification loss L(c) and an unsupervised feature reconstruction loss L(u), with L = 0.8 \times L(c) + 0.2 \times L(u); Output layer: a one-dimensional risk score (Risk Score) to quantify the probability of p53 mutation status. The network was optimized using the Adam algorithm with an initial learning rate of 0.01. The study cohort was divided into training (n = 120) and validation (n = 52) cohorts at a ratio of 7:3.

The selected radiomics features were visualized and interpreted using SHapley Additive exPlanations (SHAP) technology. SHAP values indicate the importance of each feature and its impact on model prediction probabilities, providing interpretability for the radiomics model. After training, the output risk score (RS) served as the risk value of the deep network model.

**Figure 2:**
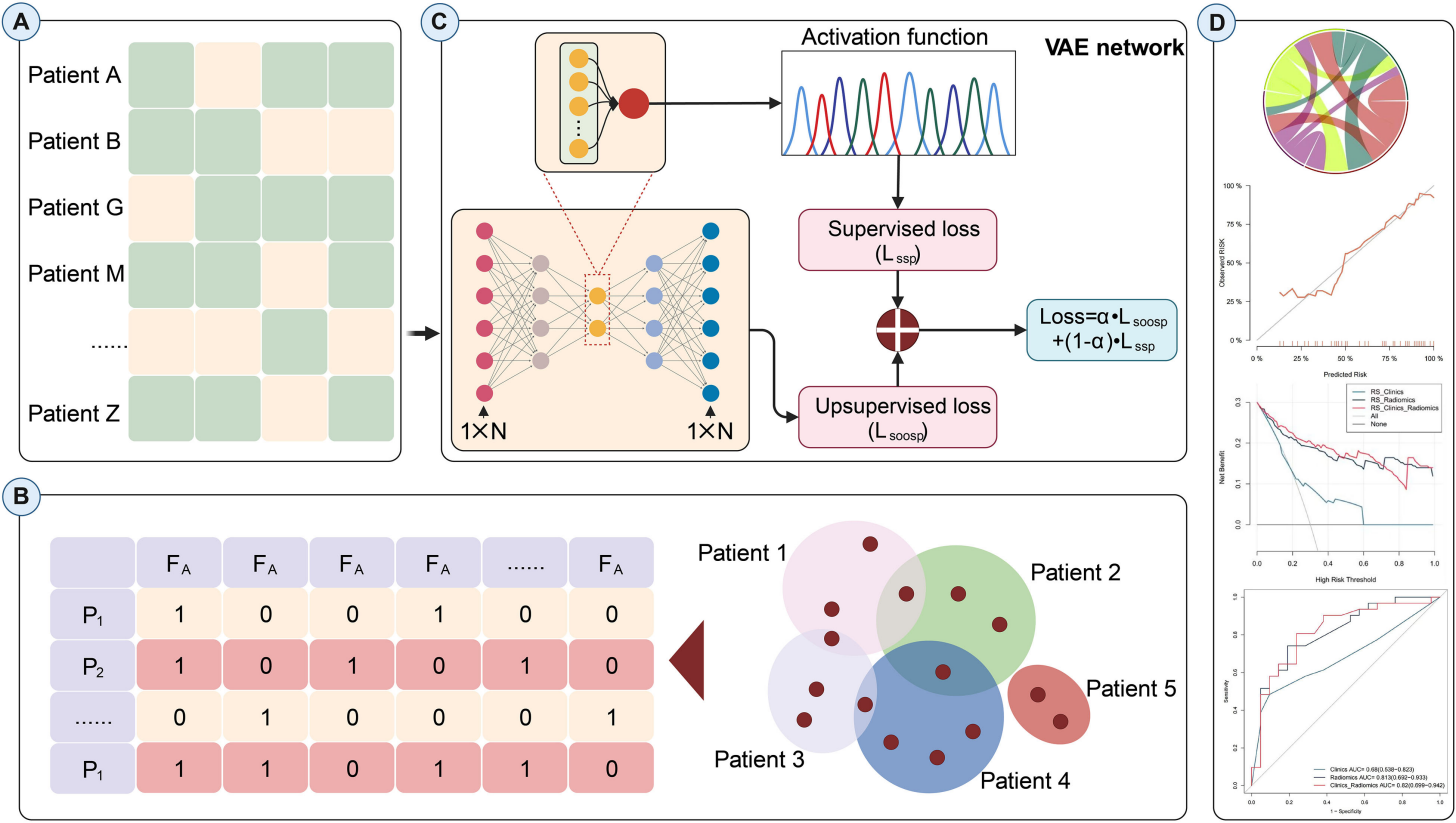
Flowchart of the ultrasound radiomics prediction model based on the variational autoencoder network.

Subsequently, the risk score derived from the VAE was combined with clinically significant variables to develop a nomogram for visualizing the model. The workflow for model construction is detailed in **Figure 2**.

### Statistical analysis

All analyzes were conducted using R statistical software (version 4.4.2; R Foundation for Statistical Computing). Continuous variables were assessed for normality using Kolmogorov-Smirnov tests and presented as mean ± standard deviation or median (interquartile range) as appropriate. Group comparisons were performed using Student’s t-test (normally distributed data) or Mann-Whitney U test (non-parametric data), while categorical variables were analyzed with Pearson’s chi-square test or Fisher’s exact test. Model discrimination was quantified through receiver operating characteristic (ROC) curve analysis. Diagnostic accuracy metrics, including sensitivity, specificity, and accuracy, were derived from optimal cutoff points determined by Youden’s index. The clinical net benefit of prediction models was systematically evaluated through decision curve analysis (DCA) across threshold probabilities ranging from 10% to 90%. Comparative model improvement was further quantified using net reclassification improvement (NRI) and integrated discrimination improvement (IDI) indices. Multidimensional associations among clinical parameters, radiomics scores, and p53 status were visualized via a nomogram.

### Ethics approval and consent to participate

This retrospective study was approved by the Medical Ethics Committee of Zhejiang Provincial People’s Hospital (No. 2025QT045) and in conformity to the Declaration of Helsinki. The requirement of informed consent was waived for this retrospective study by the Medical Ethics Committee of Zhejiang Provincial People’s Hospital based on the retrospective nature of the study.

## Results

### Patient characteristics

The study cohort comprised 172 consecutive patients (median age: 59.0 years; age range: 26–84 years) with histopathologically confirmed HCC, stratified into the p53-wild-type group (*n* = 68) and the p53-mutated group (*n* = 104). A pronounced male predominance was observed (154 males [89.5%] vs. 18 females [10.5%]). Through stratified randomization (7:3 ratio) balanced for p53 status and age, participants were allocated into training (*n* = 120; median age: 59.1 years; 12 females) and validation (*n* = 52; median age: 53.8 years; 6 females) cohorts.

Baseline clinic radiologic characteristics, including tumor staging and differentiation grade, are comprehensively detailed in **Table 1**. Comparative analysis via χ² tests (categorical variables) and Mann-Whitney *U* tests (continuous variables) confirmed no statistically significant inter-cohort differences in demographic or disease-specific parameters (all *p* > 0.05), validating the robustness of the data partitioning methodology.

**Table 1:** Clinical information of patients in training and validation cohorts.

Characteristics	Overall	Training cohort	Validation cohort	*P*-value
n	172	120	52	
MVI, n (%)				0.316
M0	81 (47.1)	53 (44.2)	28 (53.8)	
M1+M2	91 (52.9)	67 (55.8)	24 (46.2)	
Gender, n (%)				0.975
Female	18 (10.5)	12 (10.0)	6 (11.5)	
Male	154 (89.5)	108 (90.0)	46 (88.5)	
Age, mean (SD)	59.0 (11.2)	59.6 (11.4)	57.5 (10.8)	0.247
Edmondson-Steiner grade, n (%)				0.888
I-II	122 (70.9)	86 (71.7)	36 (69.2)	
III-IV	50 (29.1)	34 (28.3)	16 (30.8)	
BCLC, n (%)				0.756
0+A	160 (93.0)	112 (93.3)	48 (92.3)	
B	12 (7.0)	8 (6.7)	4 (7.7)	
Child_Pugh, n (%)				1.000
A	160 (93.0)	111 (92.5)	49 (94.2)	
B	12 (7.0)	9 (7.5)	3 (5.8)	
Diameter, mean (SD)	5.3 (3.5)	5.6 (3.7)	4.7 (3.1)	0.104
size, n (%)				0.194
≤5cm	98 (57.0)	64 (53.3)	34 (65.4)	
>5cm	74 (43.0)	56 (46.7)	18 (34.6)	
Liver_cirrhosis, n (%)				0.402
Absent	106 (61.6)	71 (59.2)	35 (67.3)	
Present	66 (38.4)	49 (40.8)	17 (32.7)	
HBsAg-/+, mean (SD)	0.8 (0.4)	0.7 (0.4)	0.8 (0.4)	0.335
Tumor Capsule, n (%)				0.693
Complete	107 (62.2)	73 (60.8)	34 (65.4)	
Incomplete	65 (37.8)	47 (39.2)	18 (34.6)	
Tumor_margin, n (%)				0.819
Smooth	92 (53.5)	63 (52.5)	29 (55.8)	
Non-smooth	80 (46.5)	57 (47.5)	23 (44.2)	
Intratumoral_necrosis, n (%)				0.129
Absent	60 (34.9)	37 (30.8)	23 (44.2)	
Present	112 (65.1)	83 (69.2)	29 (55.8)	
AFP, n (μg/L)				0.249
≤20	73 (42.4)	47 (39.2)	26 (50.0)	
>20	99 (57.6)	73 (60.8)	26 (50.0)	
TBIL, mean (SD)	18.4 (9.8)	18.7 (7.9)	17.5 (13.2)	0.538
ALB, mean (SD)	38.9 (4.5)	38.9 (4.4)	38.9 (4.7)	0.981
PT, mean (SD)	12.2 (1.0)	12.2 (1.0)	12.3 (1.1)	0.538

### Dimensionality reduction and clinical feature selection

Seventeen clinicopathological variables were initially evaluated through logistic regression (glmnet package v4.1–8 in R. The regularization path analysis identified three non-zero coefficient predictors at optimal λ: MVI status, Edmondson-Steiner histological grade, and serum AFP levels. The retained variables demonstrated significant prognostic associations in multivariable logistic regression (Wald test p < 0.01):

A clinical risk score (CRS) was computed using the linear predictor of the final model:

CRS = -0.55 + 0.80 × MVI + 0.79 × Edmondson-Steiner grade + 0.63 × AFP.

### Predictive model development for p53 status

**Figure 3:**
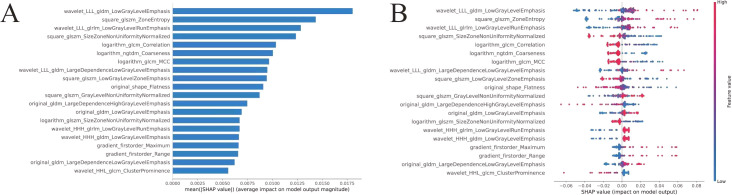
VAE selects US radiomics features and weight maps for each feature. **(A)** SHAP-values of various US features selected by VAE. SHAP-values indicate the impact of each feature on the model output (predicted results) relative to a baseline (usually the overall average predicted result), explaining the contribution of each feature to the model output. **(B)** Weight plots of the 20 US radiomics features selected by VAE.

Three distinct predictive frameworks were systematically developed: (1) Clinical Model: Derived from LASSO-selected clinicopathological variables (MVI, Edmondson grade, AFP), (2) Radiomic Model: Constructed using 20 VAE-optimized US imaging biomarkers (**Figure 3A** The detailed parameters are described in detail in the **Supplementary Data S1**), (3) Integrated Model: Concatenation-based late fusion architecture combining clinical and radiomic predictors. The VAE radiomic model identified 20 discriminative features through latent space disentanglement (The formulae of the logistic regression model in predictive models are described in detail in the **Supplementary Data S2**), predominantly comprising wavelet-transformed texture features (6/20, 30%) and square-size zone matrix descriptors (4/20, 20%). Feature-wise contributions were quantified via SHapley Additive exPlanations (SHAP; Python SHAP v0.44.0), with force plots (**Figure 3B**) revealing nonlinear relationships between radiomic patterns and proliferation status.

### Model performance evaluation and interpretability

**Figure 4:**
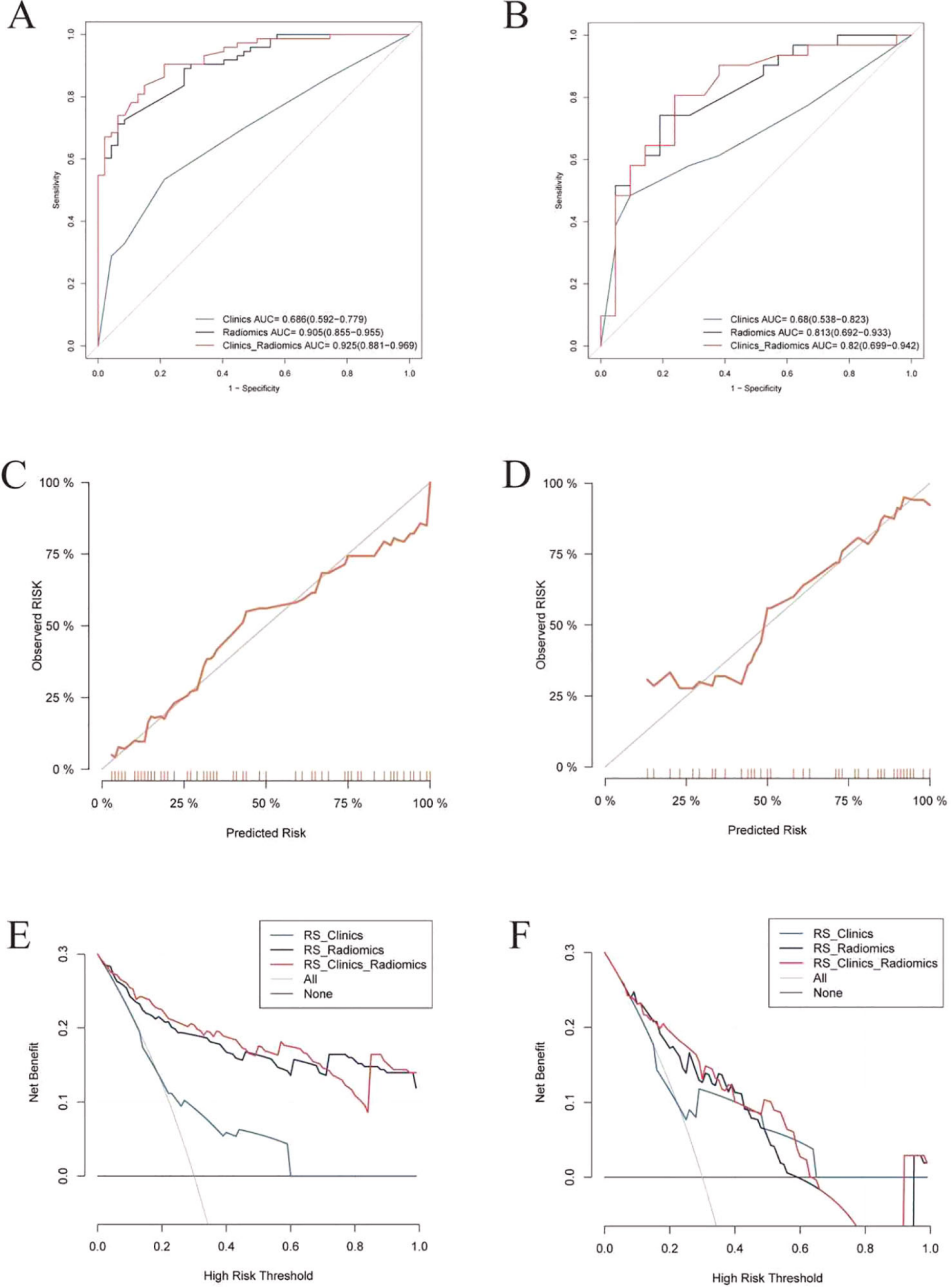
ROC, calibration, and DCA curves for all models. Participants were divided into training **(A, C, E)** and validation **(B, D, F)** cohort. **(A–B)** ROC curves for the training and validation sets. **(C–D)** Calibration curves for the training and validation sets. **(E–F)** DCA curves for the training and validation sets.

**Figure 5:**
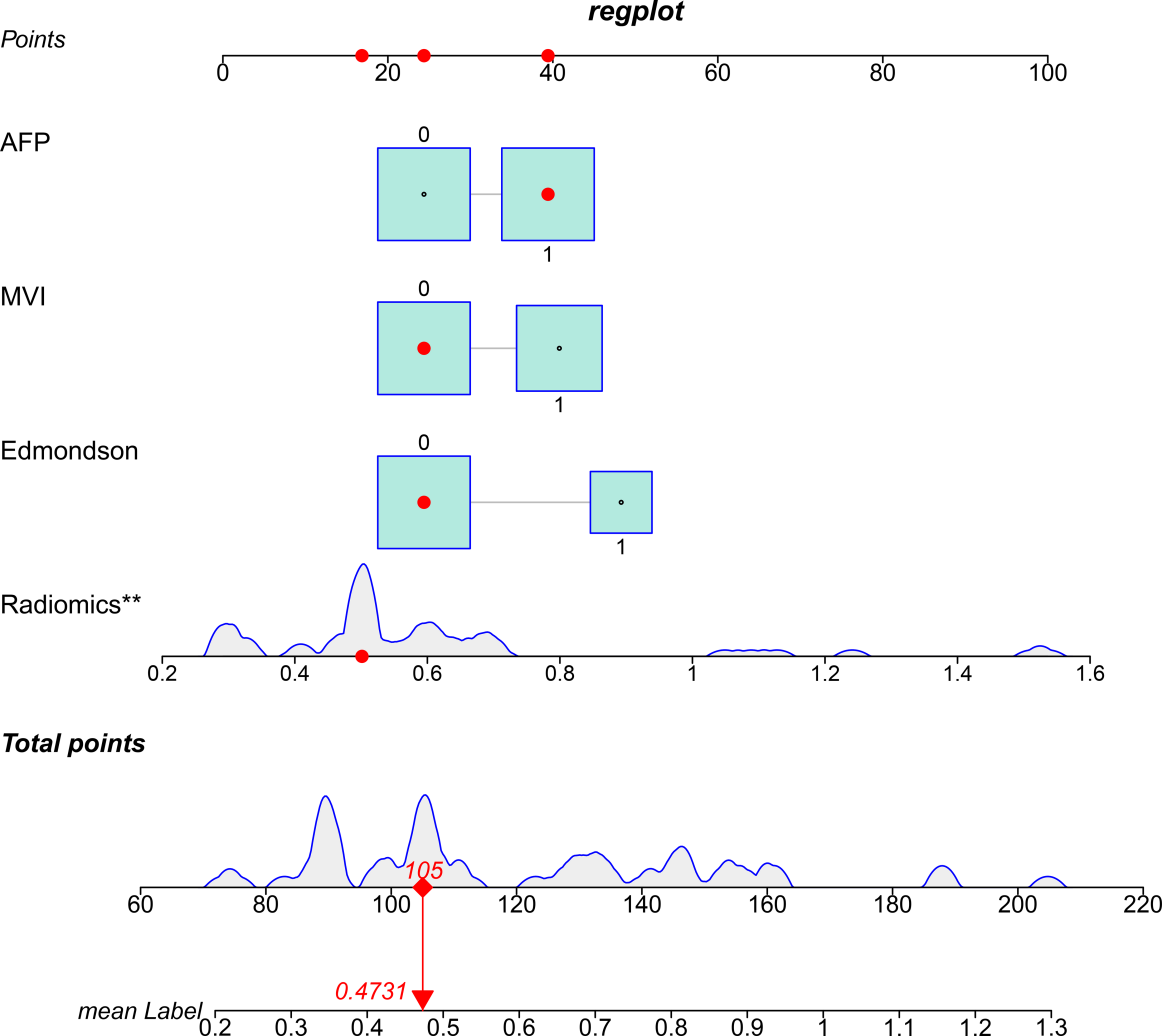
Visualization of the integrated model and analysis of variable correlations.

The prognostic performance of the clinical, radiomic, and integrated models was rigorously evaluated through validation. ROC analysis demonstrated incremental discriminative capacity: Training cohort (**Figure 4A**): Clinical model: AUC 0.69 (95% CI 0.59–0.78), Radiomics model: AUC 0.91 (0.86–0.96), Integrated model: AUC 0.93 (0.88–0.97).Validation cohort (**Figure 4B**): Clinical model: AUC 0.68 (0.54–0.82), Radiomics model: AUC 0.81 (0.69–0.93), Integrated model: AUC 0.82 (0.70–0.94). Calibration analysis revealed excellent agreement between predicted probabilities and observed outcomes, evidenced by: Hosmer-Lemeshow goodness-of-fit *p* > 0.15 across all models. (**Figures 4C, D**). Decision curve analysis (**Figures 4E, F**) confirmed the superior net benefit of the integrated model across clinically relevant threshold probabilities (15%-65%). The integrated discrimination improvement (IDI) analysis quantified significant predictive gains: IDI = 0.142 (95% CI 0.013–0.271; *p* = 0.031) vs clinical model, IDI = 0.048 (95% CI 0.005–0.090; *p* = 0.028) vs radiomics model. The final nomogram (**Figure 5**) operationalizes model outputs through point-scale conversion of SHAP-weighted predictors.

To visually represent the integrated model and analyze the relationships among various variables, we developed a nomogram integrating AFP, MVI, Edmondson-Steiner grade, and radiomics features. This nomogram is designed to predict the risk of p53 mutation in HCC patients. By combining these key clinical and radiomics features, the nomogram provides a comprehensive and intuitive tool for risk assessment.

## Discussion

This investigation establishes that a US radiomics prediction model based on a VAE network can effectively distinguish HCC patients with p53 mutation. The model exhibited high AUC values in both the training (AUC = 0.925) and validation (AUC = 0.82) cohorts. The VAE network encodes high-dimensional radiomics data into low-dimensional information through its encoder, capturing the core features of HCC imaging data for subsequent analysis tasks. By calculating risk scores from US radiomics features using the VAE network, this study shows that US radiomics can extract biological information related to p53 mutation from tumors. The integration of clinical factors (such as AFP, Edmondson-Steiner grade, and MVI) with radiomics features further enhances model performance, potentially providing a reference for personalized HCC treatment.

p53, a master tumor suppressor protein, plays a pivotal role in regulating the cell cycle and apoptosis, serves as a critical indicator of tumor cell proliferative activity. Substantial evidence underscores its pivotal role in tumorigenesis, disease progression, metastatic potential, and clinical prognosis across various malignancies (10–12). Notably, our findings corroborate demonstrating a significantly higher prevalence of p53 mutations in patients with microvascular invasion (MVI) compared to those without MVI. Furthermore, Previous studies have also shown a positive correlation between AFP levels and p53 mutations, which aligns with our results (5). While conventional p53 assessment through invasive tissue biopsies remains limited by procedural risks and sampling variability (13, 14), the advent of radiomics offers a paradigm shift in preoperative evaluation. This quantitative imaging analysis approach enables non-invasive prediction of molecular profiles, particularly advantageous for surgical ineligible patients seeking alternative diagnostic pathways.

Recent studies have advanced HCC prognosis prediction: Tang et al. proposed a dynamic graph network to predict TACE therapy progression (15), while quantitative MRI markers have shown efficacy in non-invasively predicting molecular markers like PD-L1 (5). This study leverages clinically accessible and noninvasive two-dimensional ultrasound imaging to develop a predictive model with favorable clinical applicability. By integrating deep learning techniques with radiomics, we employed a variational autoencoder (VAE) to automatically extract high-dimensional latent features from ultrasound images (16), overcoming the limitations of manual feature selection and enhancing both the comprehensiveness and sensitivity of feature representation. Furthermore, we incorporated deep learning-derived imaging features with established clinical parameters (including alpha-fetoprotein [AFP] levels and microvascular invasion [MVI] status) to construct a comprehensive nomogram, which synergistically combines imaging biomarkers with routinely available clinical variables to improve predictive performance and clinical utility. Rigorous methodology including training-validation dataset partitioning and multifaceted validation (with receiver operating characteristic [ROC] curve analysis, calibration assessment, and decision curve analysis) ensured robust model accuracy and generalizability, ultimately establishing a reliable noninvasive tool for assessing p53 mutation status in clinical practice.

The primary limitations of this study stem from its single-center retrospective design, although the VAE framework helps mitigate overfitting, the sample size (*n* = 172) is still relatively modest for deep learning research. The reliance on two-dimensional ultrasound imaging, characterized by relatively limited spatial resolution and suboptimal depiction of three-dimensional tumor architecture, may have resulted in the omission of subtle features within deep or small lesions, potentially compromising the completeness of feature extraction. While the predictive performance was rigorously evaluated through internal validation, the absence of external validation across diverse patient populations necessitates further verification in multi-institutional cohorts to confirm model robustness and broader clinical applicability.

## Conclusions

This study demonstrates that VAEs provide an effective framework for addressing the high-dimensional complexity inherent in radiomics data analysis. The VAE-driven radiomics framework achieving superior predictive performance (AUC: 0.925 training, 0.820 validation), providing a reliable tool for noninvasive preoperative evaluation of HCC p53 mutation. The model shows promise for optimizing surgical decision-making and personalized prognostic strategies in HCC management.

## Data Availability

The original contributions presented in the study are included in the article/**Supplementary Material**. Further inquiries can be directed to the corresponding authors.
