# Supplementary material for: Ultrasound - based deep learning radiomics nomogram for noninvasive prediction of p53 mutation status in hepatocellular carcinoma: a variational autoencoder based development and validation study
Source: Front Oncol. 2026 Apr 29;16:1756941. doi: 10.3389/fonc.2026.1756941 (PMC13167416; doi:10.3389/fonc.2026.1756941)
Supplement: Supplementary file 1 [file Table1.docx]

**Supplementary Materials**

| **Supplementary Data S1.** Radiomic features retained after VAE screening |
| --- |
| \| Features \| \| --- \| \| 1. wavelet_LLL_gldm_LowGrayLevelEmphasis  2. square_glszm_ZoneEntropy  3. wavelet_LLL_glrlm_LowGrayLevelRunEmphasis  4. square_glszm_SizeZoneNonUniformityNormalized  5. logarithm_glcm_Correlation  6. logarithm_ngtdm_Coarseness  7. logarithm_glcm_MCC  8. wavelet_LLL_gldm_LargeDependenceLowGrayLevelEmphasis  9. square_glszm_LowGrayLevelZoneEmphasis  10. original_shape_Flatness  11. square_glszm_GrayLevelNonUniformityNormalized  12. original_gldm_LargeDependenceHighGrayLevelEmphasis  13. original_gldm_LowGrayLevelEmphasis  14. logarithm_glszm_SizeZoneNonUniformityNormalized  15. wavelet_HHH_glrlm_LowGrayLevelRunEmphasis  16. wavelet_HHH_gldm_LowGrayLevelEmphasis  17. gradient_firstorder_Maximum  18. gradient_firstorder_Range  19. original_gldm_LargeDependenceLowGrayLevelEmphasis  20. wavelet_HHL_glcm_ClusterProminence \| |

**Supplementary Data S2.** **The formulae of the logistic regression model in predictive models**

The formula of all model using Logistic model was as following:

（A） clinical risk score (CRS) was computed using the linear predictor of the final model: CRS = -0.55+0.80 × MVI + 0.79 × Edmondson + 0.63 × AFP

(B)Integrated model risk score(IRS):

IRS = -7.40+1.50 × MVI + -0.12 × Edmondson + 0.55 × AFP+13.18×Radiomics
